# The Asymptotic Noise Distribution in Karhunen-Loeve Transform Eigenmodes

**DOI:** 10.4172/2157-7420.1000122

**Published:** 2013-06

**Authors:** Yu Ding, Hui Xue, Ning Jin, Yiu-Cho Chung, Xin Liu, Yongqin Zhang, Orlando P. Simonetti

**Affiliations:** 1 Davis Heart and Lung Research Institute, The Ohio State University, Columbus, USA; 2 Shenzhen Institute of Advanced Technology of Chinese Academy of Science, Shenzhen, Guangdong, China; 3 Siemens Corporate Research, Princeton, USA; 4 Siemens Medical Solutions, Inc., Columbus, USA; 5 Department of Internal Medicine, The Ohio State University, Columbus, USA; 6 Department of Biomedical Engineering, The Ohio State University, Columbus, USA; 7 Department of Radiology, The Ohio State University, Columbus, USA

**Keywords:** Karhunen-Loeve transform, Random matrix theory, Independently and identically-distributed noise

## Abstract

Karhunen-Loeve Transform (KLT) is widely used in signal processing. Yet the well-accepted result is that, the noise is uniformly distributed in all eigenmodes is not accurate. We apply a result of the random matrix theory to understand the asymptotic noise distribution in KLT eigenmodes. Noise variances in noise-only eigenmodes follow the Marcenko-Pastur distribution, while noise variances in signal-dominated eigenmodes still follow the uniform distribution. Both the mathematical expectation of noise level in each eigenmode and an analytical formula of KLT filter noise reduction effect with a hard threshold were derived. Numerical simulations agree with our theoretical analysis. The noise variance of an eigenmode may deviate more than 60% from the uniform distribution. These results can be modified slightly, and generalized to non-IID (independently and identically-distributed) noise scenario. Magnetic resonance imaging experiments show that the generalized result is applicable and accurate. These generic results can help us understand the noise behavior in the KLT and related topics.

## Introduction

Karhunen-Loeve Transform (KLT, a.k.a. principal component analysis or singular value decomposition) is an important tool in dimension reduction, data mining, and denoising [1,2]. It transforms the original data set into a series of orthogonal eigenmodes with eigenvalues *λ*_1_ ≤ *λ*_2_ ≤ … ≤ *λ*_m_. It is believed that when a data set is corrupted by additive independently and identically-distributed (IID) noise with variance σ^2^, the unitarity of the KLT ensures that the noise in each eigenmode is also white with variance σ^2^ [3,4,5]. Based on this assumption, the noise variance can be estimated from the smallest eigenvalue *λ*_1_ [6]. Furthermore, a threshold can be applied safely to remove all noise-only eigenmodes, eg. truncate all eigenmodes with λi ≤ σ^2^ (assume σ^2^ is known) in order to suppress noise using a KLT filter [5,7].

However, the noise variance in KLT eigenmodes may not be uniformly distributed. When noise-only eigenmodes appear, a theoretical result of the random matrix theory (RMT) [8] predicts that the corresponding eigenvalues follow the Marcenko-Pastur (MP) distribution [9,10], instead of the uniform distribution. Therefore, the “common sense” of the noise distribution in KLT eigenmodes mentioned above may not be accurate enough. In this communication, we apply this RMT result to study the noise variance distribution in KLT eigenmodes. We show that the variances in noise-only eigenmodes follow the MP distribution, while noise variances in signal-dominated eigenmodes still follow the uniform distribution. In addition, the smallest eigenvalue *λ*_1_ could be much lower than σ^2^, and the eigenvalues of some noise-only eigenmodes may be much higher than σ^2^. Based on this result, first, we derive the mathematical expectation of the noise level in each eigenmode; second, we derive an analytical relation between the threshold or filter cutoff and the noise level in the KLT filtered data set. However, IID noise is rarely the case in practice. Noise usually has some degree of correlation and amplitude variation. Therefore, MP distribution is not applicable. According to our previous study, the MP distribution can be modified slightly by re-define a parameter, and become applicable to some important non-IID noise scenario [11]. Numerical simulations are used to validate our results, and real-time MR cardiac cine images are used to show that our results can be utilized in experimental data analysis. Therefore, our research helps clarify theoretically the noise reduction effect of KLT-based filter used in medical imaging society [5,7], and provides an explanation why the random matrix theory based noise level estimation [11] is better than smallest eigenvalue based method [6].

## Theory

### Additive independently and identically-distributed noise

We assume that a data set can be represented by an *m* by *n* matrix *A*, without loss of generality, *m*<*n*. ***A*** can be regarded as a sum of a low rank signal matrix ***S*** and a random matrix ***N*** with IID noise (variance=σ^2^) in each entry:

(1) A=S+N

The rank of ***S*** is *r*, and *r*<*m*. In other words, there is intrinsic redundancy in ***S*** and ***A***. The empirical covariance matrix ***R*** of ***A***, defined as ***AA^T^***/*n* (***A^T^*** represents the transpose of ***A***) is full rank. When the smallest non-zero eigenvalue of the covariance matrix of S satisfies *λ*_min_≥ σ^2^√(*d/n*), the Probability Distribution Function (PDF) of the smallest *d* (where *d*=*m*-*r*) eigenvalues of ***R*** follows the Marcenko-Pastur (MP) distribution from the RMT [9,10]:

(2) $$p(\lambda )=\frac{1}{2\pi \alpha \sigma^{2}\lambda }\sqrt{\max(0,(\lambda_{+}-\lambda )(\lambda -\lambda_{-}))},$$

Where *α*=*d/n*, $\lambda_{\pm }=\sigma^{2}{\left(1\pm \sqrt{\alpha }\right)}^{2}$. Equation 2 is an asymptotic result when *n,d* ≫*1* (from our experience *n,d*>10 is sufficient, in agreement with previously published observations [12]. The first d eigenmodes are noise-only (eigenmodes are sorted in ascending order by eigenvalues); eigenvalues *λ*_i_ (*i*=1, …, *d*) are the noise variances of the corresponding eigenmodes. Therefore, the noise variance PDF of the first d eigenmodes follows equation 2. Please refer to equation A.2 in Appendix for the mathematical expectation of the noise level in each eigenmode. The smallest eigenvalue *λ*_1_ is lower than σ^2^. When α~1, *λ*_1_ is a poor estimation of *σ*^2^ (*λ*_1_≪σ^2^).

The last *r* eigenmodes are signal-dominated. Because signal is uncorrelated with noise and due to the unitarity of KLT, noise variances are identical (=σ^2^) in the last *r* eigenmodes. Therefore, noise variances in first *d* eigenmodes follow the MP distribution, while noise variances in last *r* eigenmodes follow the uniform distribution.

The KLT filter can be applied by using a threshold to truncate some eigenmodes, and then reconstructing the filtered matrix ***Â*** using the remaining eigenmodes. Therefore, ***Â*** is a low-rank approximation of ***A***, and is optimal in the 2-norm sense [5,13]. The noise variance distribution in eigenmodes can describe the noise variance in ***Â*** when the eigenvalue threshold=*λ*_c_. Suppose *k* eigenmodes with eigenvalues ≤ *λ*_c_ are truncated. The mean noise variance *f*(*k*) of KLT filtered ***Â*** is:

(3) $$f(k)=\frac{1}{m}\sum_{i}{\hat{\sigma }}_{i}^{2}=\frac{m\sigma^{2}-h(k)}{m}$$

where ${\hat{\sigma }}_{i}^{2}$ is the mean noise variance of the *i*^th^ row of ***Â***; *h*(*k*) is the total noise variance in k truncated eigenmodes. In order to deduce Equation 3, we take advantages of two properties: first, the KLT is unitary; second, the noise variance is additive. When *λ_c_* ≥ *λ*_+_,

(4a) $$h(k)=k\sigma^{2}$$

When *λ_c_*<*λ*_+_,

(4b) $$h(k)=\frac{k}{4\pi \alpha \sigma^{2}}\{\left(\lambda_{c}-\frac{\lambda_{-}+\lambda_{+}}{2}\right)\sqrt{{\left(\frac{\lambda_{+}-\lambda_{-}}{2}\right)}^{2}-{\left(\lambda_{c}-\frac{\lambda_{-}+\lambda_{+}}{2}\right)}^{2}}+{\left(\frac{\lambda_{+}-\lambda_{-}}{2}\right)}^{2}\arcsin\frac{2\lambda_{c}-(\lambda_{-}+\lambda_{+})}{\lambda_{+}-\lambda_{-}}\}+\frac{k\sigma^{2}}{2}$$

Please refer to the Appendix for more details.

When α→0, p(*λ*)→δ(*λ*−σ^2^), (4b) is identical to (4a), and (3) becomes:

(5) $$f_{0}(k)=\lim_{\alpha \to 0}\;f(k)=\frac{m-k}{m}\sigma^{2}$$

### Additive Non-IID Noise

The MP-law is only applicable to additive IID noise in rank deficit data matrix ***A***. If matrix ***N*** is non-IID additive noise, then the noise covariance of matrix ***N*** become a fourth order tensor ***C***_ij,kl,_ where (i, j) and (k, l) are indices of two data points in matrix ***A***. If the noise is IID, tensor ***C***_ij,kl_ degenerates into the multiplication of two identity matrices: *σ*^2^*δ*_ij_*δ*_kl_, where *σ*^2^ is the noise variance, and *δ*_ij_ is the Kronecker delta. Our recent study found that the MP-law is still applicable when the noise covariance tensor can be written as: ***D***_ij_*δ*_kl_, where ***D*** is a generic 2-D covariance matrix. In other words, the noise is IID only in one dimension. In practice, this is a very common scenario when the noise has spatial correlations but no temporal correlations, such as Magnetic Resonance (MR) dynamic imaging. It has been shown that a modified MP-law is still applicable in dynamic MR images, i.e. equation 2 is still accurate enough with modified parameters: *n*→*n*′, *α*→*α*′=*d/n*′, $\lambda_{\pm }\to \lambda_{\pm }^{\prime }=\sigma^{2}{\left(1\pm \sqrt{\alpha^{\prime }}\right)}^{2}$ [11]. Hence, the rest of the equations are also applicable because they are derived from equation 2. Please note that the application of modified MP distribution to non-IID noise scenario is only an empirical approximation, a strict mathematical proof is warranted.

## Methods

We tested the effectiveness of equations 2–4, using numerical simulations and real-time dynamic MR cardiac imaging in a volunteer. The human study was approved by The Ohio State University’s Human Subjects Committee and all subjects gave written informed consent to participate. The volunteer images were acquired on a 3.0T MRI system (MAGNETOM Trio, Siemens Healthcare Inc., Erlangen, Germany). A 32-channel cardiac array coil (*In vivo*, Gainesville, FL) was used for data acquisition. All data was processed using Matlab^®^ 2011a (MathWorks, Natick, Massachusetts) running on a personal computer with Intel^®^ Core(TM)2 Quad 3.0 GHz CPU, 16 GB system RAM.

A numerical model was constructed to simulate a dynamic image series with temporal redundancy. The model consisted of a bright circle on a dark background [5]. A series of 256 images was synthesized, each image having 64×64 pixels (i.e. *m*=256, *n*=64×64 in data matrix ***A***, with columns representing temporal samples and rows representing spatial samples). The circle diameter was varied sinusoidally through 29 unique radii with step size=1.0 pixel (*r*=29, *d*=227, *α*=227/(64×64) in equation 2). The smallest non-zero eigenvalue of the temporal covariance matrix of the image series was 5.2. It was then corrupted by Gaussian IID noise with *σ*^2^=1.0.

First, the temporal KLT was applied to the simulated images series. The noise variance in each eigenmode was measured, plotted, and compared to the prediction of equation A.2. The first 227 (d) eigenmodes were noise-only. Second, the noise variance of the KLT filtered images *f*(*k*) was measured by varying the number of truncated eigenmodes *k*. The difference between equation 3 and equation 5 was plotted and compared to the theoretical results. The noise variance was measured in a region outside of the beating disk with area=21% of the entire image. All simulations were performed 400 times, and results were averaged to suppress random fluctuations.

MR real-time cardiac cine images with uniformly down-sampled temporally-interleaved k-space were reconstructed using parallel MR technique TGRAPPA [14]. We acquired three SSFP real-time cine image series using TGRAPPA with parallel acceleration rates=5, in vertical and horizontal long-axis, and one short-axis views. Imaging parameters were: 192×95 matrix reconstructed from 192×15 acquired matrix, 6 mm thick slice, flip angle=48°, TE/TR=1.0/2.56 ms, pixel bandwidth=1447 Hz/pixel, FOV=380×285 mm^2^. A total of 256 frames were acquired per image series.

The temporal KLT was applied to the simulated images series. The number of noise-only eigenmodes and the mean noise variance were determined by maximizing the goodness-of-fit between MP-law and the smallest eigenvalues. More details of the fitting can be found in other publication [11]. The noise variance in each noise-only eigenmode was measured, plotted, and compared to the prediction of equation A.2.

## Results

**Figure 1:**
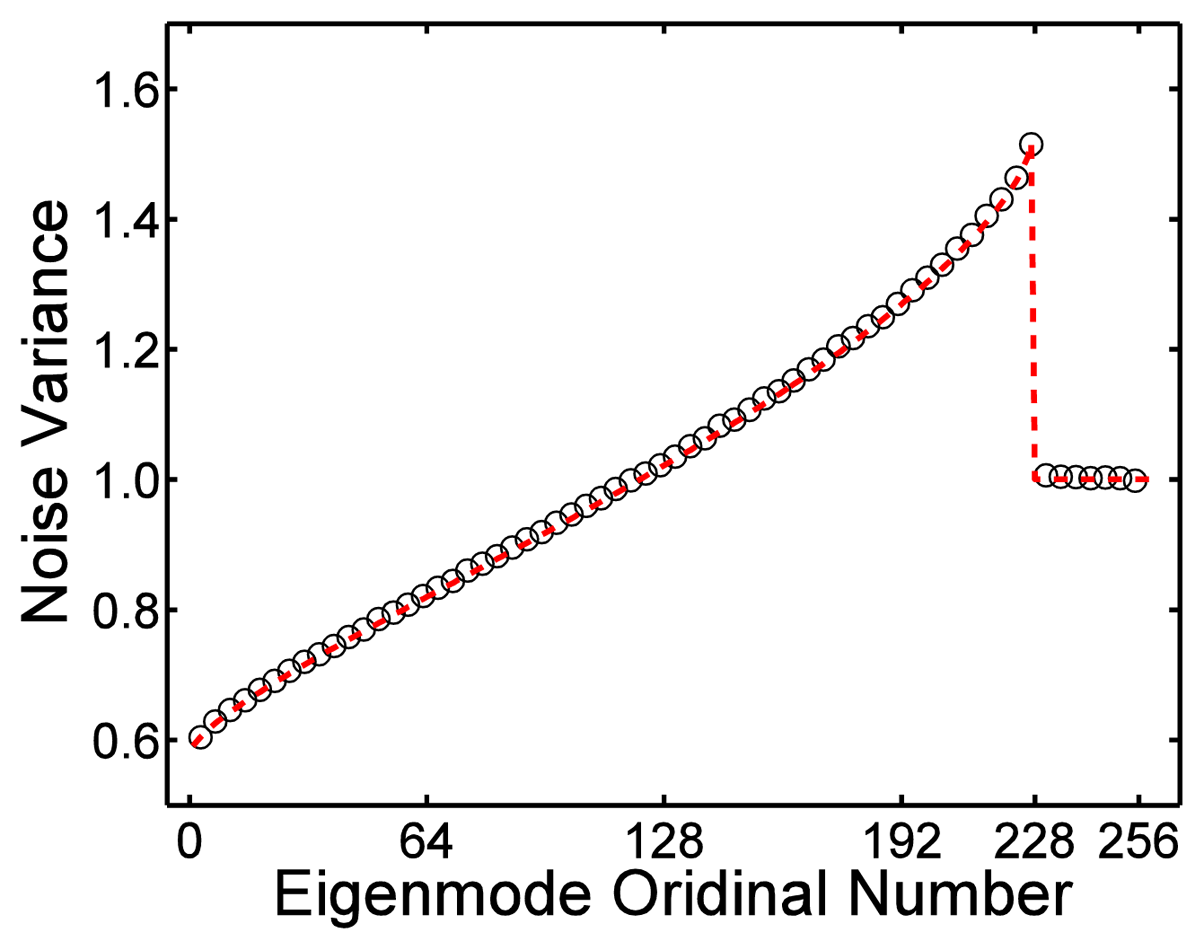
The mean noise variance measured in all eigenmodes. The solid line indicates the theoretical prediction of Equation (A.2), ○ represents the simulation result.

The mean noise variance in each eigenmode was plotted in figure 1. Notice the jump between the noise-only eigenmodes and the signal dominated eigenmodes at *λ*_227_. The highest eigenvalue=1.53, more than 50% higher than the added noise variance. Compared to the result predicted by equation 2, the maximum relative difference was less than 2.1%.

**Figure 2:**
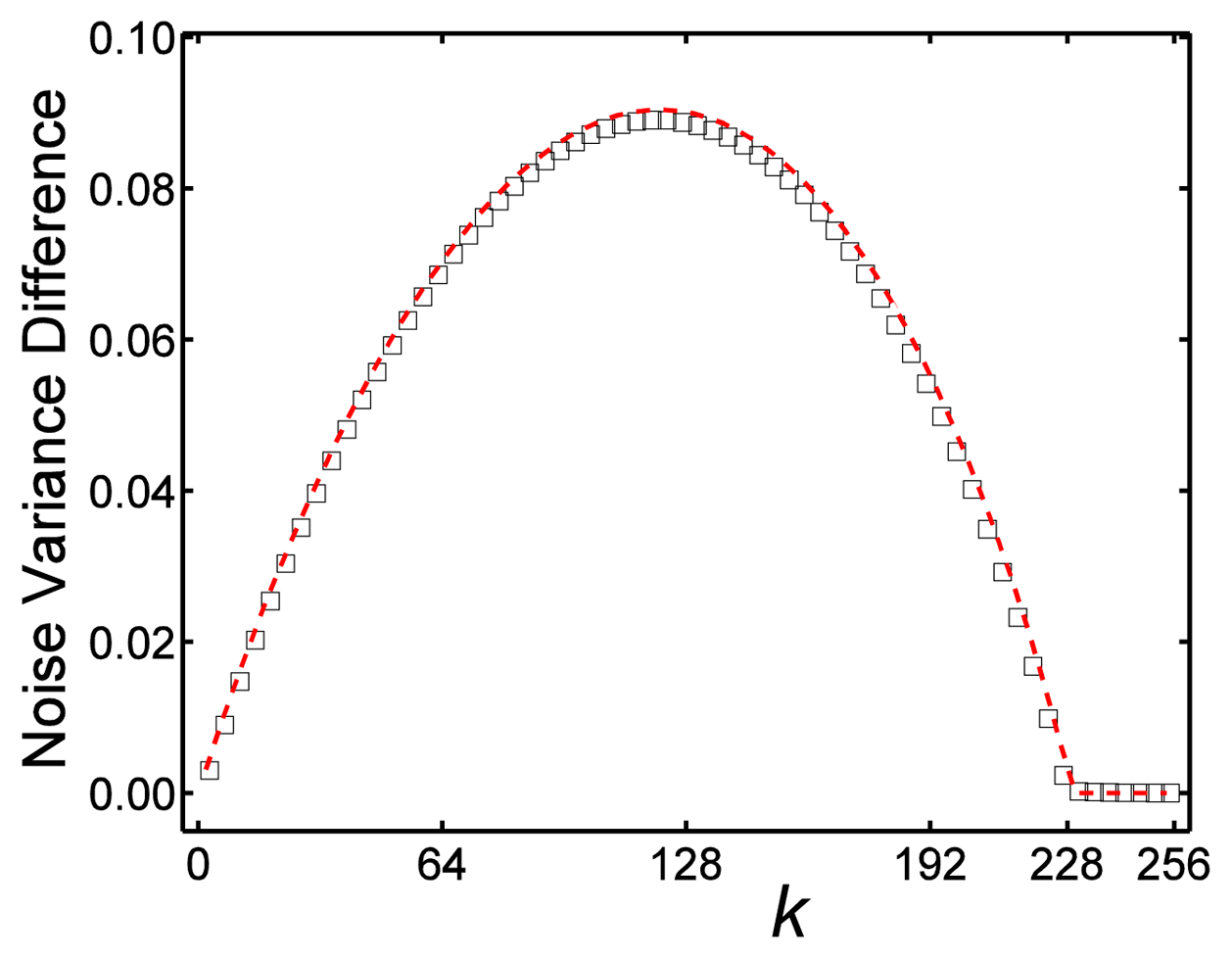
The mean noise variance difference in the KLT filtered images, *f_0_*(*k*)- *f*(*k*) vs. the number of truncated eigenmode k. The dashed line indicates the theoretical calculation from (3) & (5), ○ represents the simulation result.

The difference between the measured and predicted mean noise variance in KLT filtered images as a function of the number of truncated eigenmodes (*k*) is plotted in figure 2. The noise variance predicted by equation 5 was subtracted from the results obtained from the simulated images. The maximum deviation between the theoretically predicted results and simulation was less than 3.8% of the mean noise variance.

**Figure 3:**
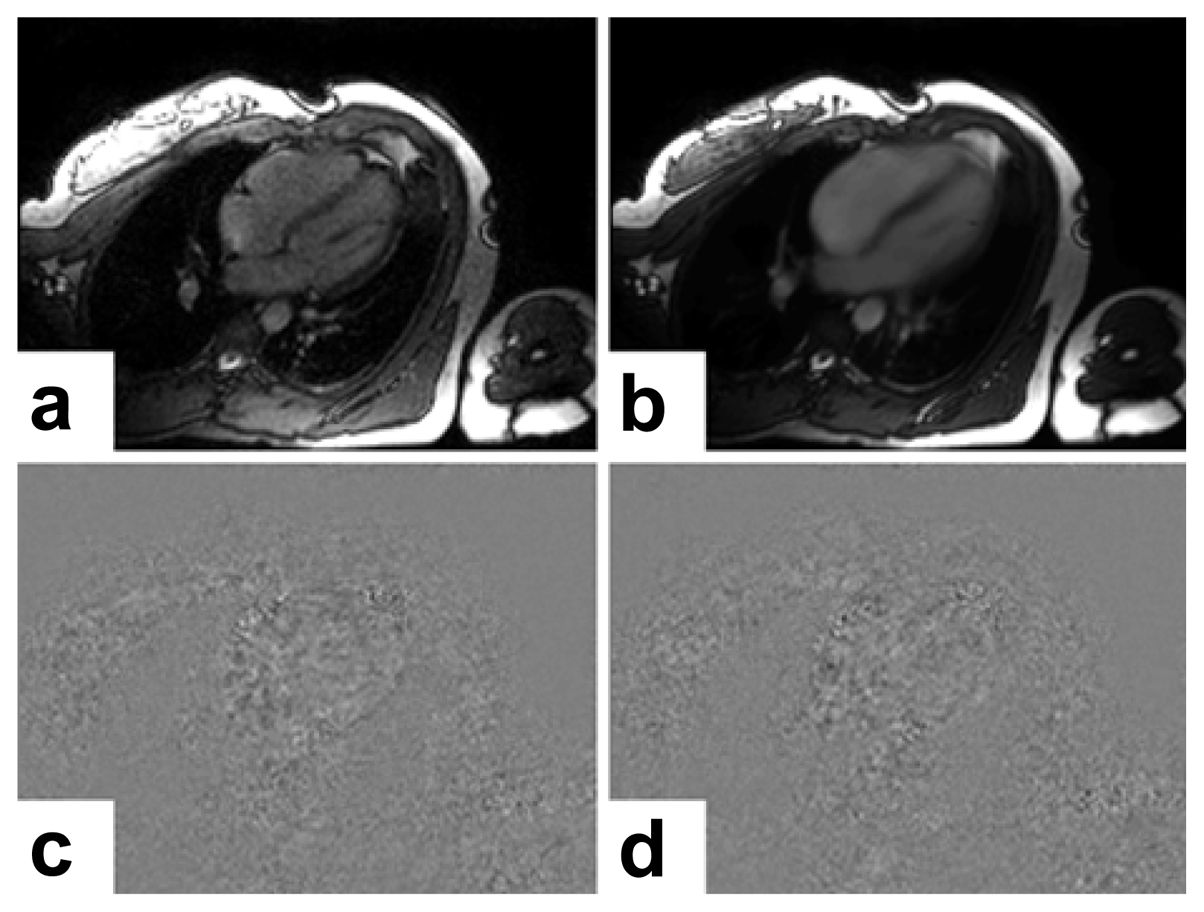
Figure 3(a). The long axis view of the heart; **(b)** the eigenmode with the largest eigenvalue; **(c)** the first signal dominate eigenmode; **(d)** the last noise-only eigenmode.

Figure 3 shows the cardiac image, and the corresponding eigenmodes used in the analysis. Figure 3c is the first eigenmode that is identified as signal dominated, and figure 3d is the last eigenmode that is identified as noise. The differences between them are too small to be distinguished by human eye easily.

**Figure 4:**
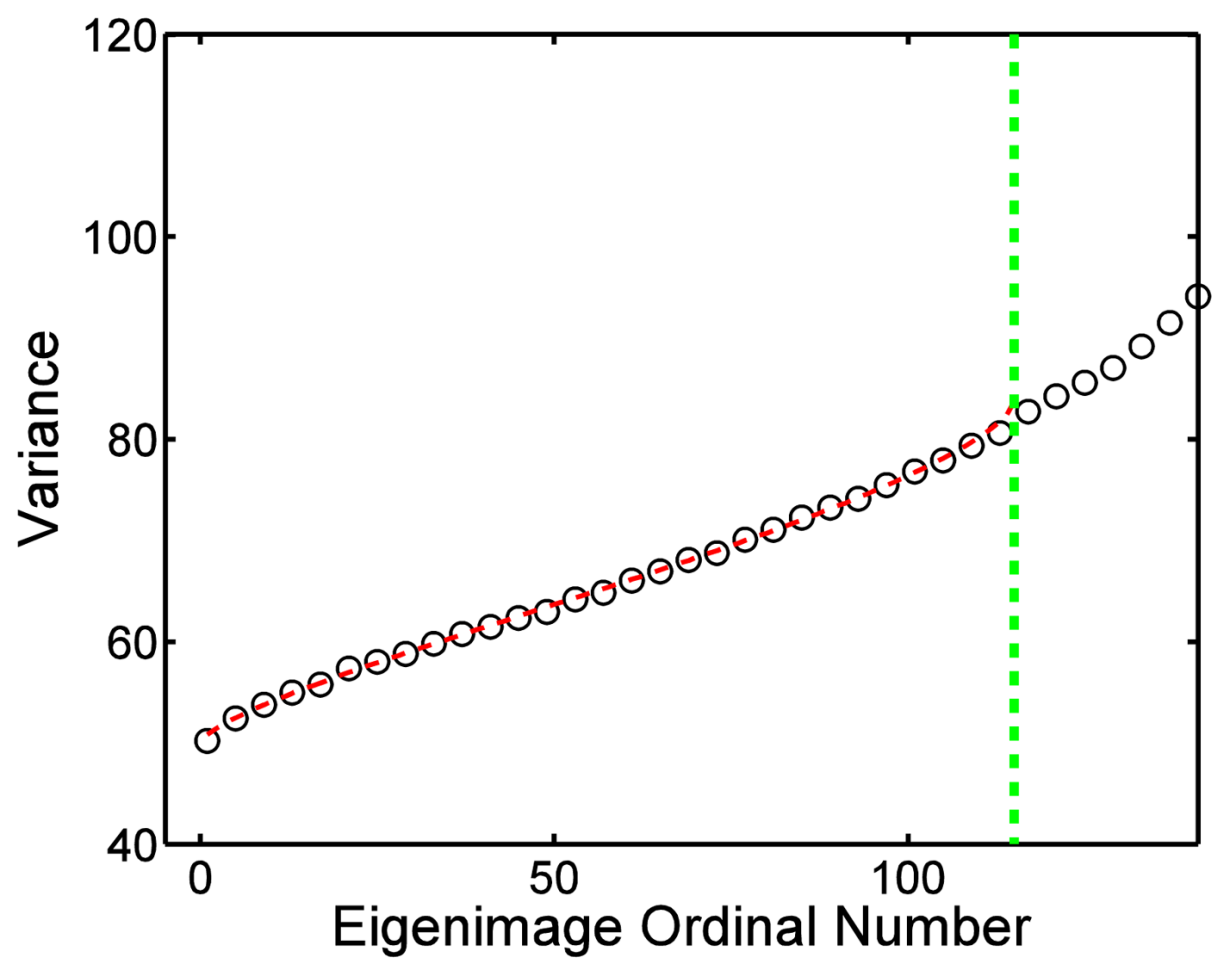
The mean noise variance measured in the first 148 eigenmodes in real-time cardiac MR cine image series, and first 115 eigenmodes were identified as noise-only. The dashed line indicates the theoretical prediction of Equation (A.2), ○ represents the experimental result. The vertical dotted line indicates the cutoff between noise-only eigenmodes and signal dominated eigenmodes.

The variance of each eigenmode of real-time MR cardiac cine image series of the short-axis view was plotted in figure. 4. Below the cutoff of noise-only eigenmodes, variance of each eigenmode follow the prediction of the modified MP distribution. The difference between the variances of the first noise-only eigenmode and the last noise-only eigenmode is as large as 63%. Again, the uniform distribution is a poor approximation of noise distribution between eigenmodes.

The smallest eigenvalues in all three real-time cardiac MR cine image series follow the MP-law with modified parameters. The ratios of *n′/n* are 0.27, 0.23, and 0.25 for three image series. Since only 20% of raw data is collect when reconstructed using parallel imaging technique with acceleration factor=5, the modified parameter n′ can be interpreted as the fitted number of independent noise samples in the image series.

## Discussion

Our results reveal the noise variance distribution among eigenmodes of the KLT, and provide a generic and accurate formula to quantify the noise reduction effect of the KLT filter with a hard threshold. Numerical simulations demonstrate that the PDF of the noise variance in noise-only eigenmodes follows equation 2, while the noise variance in signal-dominated eigenmodes is still uniformly distributed. The noise reduction effect of the KLT filter follows the prediction of equation 3 and 4 closely. When α~1, there is significant deviation from equation 5. Interestingly, when the eigenmode cutoff *k*>*d, i.e*., some of the signal-dominant eigenmodes are truncated, equation 5 is still accurate regardless of the value of α.

When matrix ***N*** in equation 1 contains two types of non-IID noise, spatially correlated noise and spatially variant noise, such as the noise in the magnetic resonance images acquired with parallel imaging techniques, the MP distribution is still valid but with a modified spatial sample number *n*′<*n*. The *n*′ can be interpreted as the “effective” independent noise samples in each column of matrix ***N***. Therefore, the noise reduction effect of the KLT filter can still be studied by the same approach. For an arbitrary data matrix ***A***, the problem of estimation parameters *r,n*′*,α* and *σ*^2^ has been solved by maximizing statistical goodness-of-fit [11]. Therefore, the noise estimation method proposed by Ready [6] systematically underestimated the noise level, and should be replaced by the more precise method [11] based on the random matrix theory.

## Conclusion

We used the RMT to study the asymptotic noise variance distribution in KLT eigenmodes, as well as the noise reduction effect of the KLT filter. These results can be used to understand the noise behavior in the KLT and related topics.
